# Estradiol and genistein effects on the sea bass (*Dicentrarchus labrax*) scales: Transcriptome dataset

**DOI:** 10.1016/j.dib.2019.104587

**Published:** 2019-10-15

**Authors:** Patricia I.S. Pinto, André R. Andrade, Michael A.S. Thorne, M. Dulce Estêvão, Adelino V.M. Canario, Deborah M. Power

**Affiliations:** a Centro de Ciencias do Mar (CCMAR), Universidade do Algarve, Campus de Gambelas, Edifício 7, 8005-139, Faro, Portugal; b British Antarctic Survey (BAS), High Cross, Madingley Road, Cambridge, CB3 0ET, UK; c Escola Superior de Saúde, Universidade do Algarve, Campus de Gambelas, Edifício 1, 8005-139, Faro, Portugal

**Keywords:** Estrogen-responsive genes, Genistein-responsive genes, Fish scale, RNA-seq, Transcriptome

## Abstract

Fish scales are mineralized structures that play important roles in protection and mineral homeostasis. This tissue expresses multiple estrogen receptor subtypes and can be targeted by estrogens or estrogenic endocrine-disrupting compounds, but their effects are poorly explored. The transcriptome data here presented support the findings reported in the research article “**Genistein and estradiol have common and specific impacts on the sea bass (*Dicentrarchus labrax*) skin-scale barrier**” [1]. Juvenile sea bass were exposed to estradiol and the phytoestrogen genistein for 1 and 5 days, by intraperitoneal injections, and the effects on scale transcript expression were analysed by RNA-seq using an Illumina Hi-seq 1500. The raw reads of the 30 libraries produced have been deposited in the NCBI-SRA database with the project accession number SRP102504. Mapping of RNA-seq reads against the sea bass reference genome using the Cufflinks/TopHat package identified 371 genes that had significant (FDR<0.05) differential expression with the estradiol or genistein treatments in relation to the control scales at each exposure time, 254 of which presented more than a 2-fold change in expression. The identity of the differentially expressed genes was obtained using both automatic and manual annotations against multiple public sequence databases and they were grouped according to their patterns of expression using hierarchical clustering and heat-maps. The biological processes and KEGG pathways most significantly affected by the estradiol and/or genistein treatments were identified using Cytoscape/ClueGO enrichment analyses.

**Table:** Specifications Table

Subject area	*Biology*
More specific subject area	*Aquaculture, Ecotoxicology, Environment, Marine fisheries*
Type of data	*Tables, figures*
How data was acquired	*Illumina Hi-Seq 1500*
Data format	*Raw, metadata*
Experimental factors	*Marine cultured sea bass were exposed to estradiol and genistein by intraperitoneal injection and their scales collected after 1 or 5 days*
Experimental features	*RNA extraction, quality evaluation, RNA-seq library preparation, sequencing and bioinformatics analysis*
Data source location	*Faro, Algarve, Portugal (37° 1′ 0″ N;7° 56′ 0″W)*
Data accessibility	*Data is available in this article and at the NCBI Sequence Read Archive (SRA), project SRP102504 (*https://www.ncbi.nlm.nih.gov/sra/SRP102504*). The links for each dataset of replicates for the six experimental groups are:*https://www.ncbi.nlm.nih.gov/sra/SRX2673957[accn*] for C1d,*https://www.ncbi.nlm.nih.gov/sra/SRX2673962[accn*] for E1d,*https://www.ncbi.nlm.nih.gov/sra/SRX2674307[accn*] for Gen1d,*https://www.ncbi.nlm.nih.gov/sra/SRX2674405*[accn] for C5d,*https://www.ncbi.nlm.nih.gov/sra/SRX2674467*[accn]* *for E5d and*https://www.ncbi.nlm.nih.gov/sra/SRX2675383*[accn]* *for Gen5d.*
Related research article	*“Genistein and estradiol have common and specific impacts on the sea bass (Dicentrarchus labrax) skin-scale barrier”* [1].

**Table:** 

**Value of the Data** • This is the first comprehensive study of the transcriptome of fish scales, an estrogen-target tissue for which information is still limited and a promising practical model for environmental pollution screening and chemical risk assessment. • The dataset has relevance for aquaculture and for toxicology/ecotoxicology/environmental studies, since altered levels of hormones (including synthetic, anthropogenic hormones) could affect the development and homeostasis of multiple marine species. • The dataset has broad applicability as it is from the European sea bass, which is a representative of the species-rich order Perciformes and an important species for marine fisheries and aquaculture. • Global transcriptomes of scales from the European sea bass, exposed for 1 and 5 days to estradiol (a natural estrogen) or genistein (a phytoestrogen) is of use for aquaculture, ichthyologists, toxicologists and comparative endocrinologists. Since estrogenic compounds and genistein can be found in aquatic environments the dataset is of use for aquaculture (e.g increased ingestion of phytoestrogens from plant-based ingredients used in fish feeds) and the comparison of genes and pathways regulated or disrupted by estradiol and genistein is of great interest for studies related to physiology, ecotoxicology/environmental studies or aquaculture in fish or other marine species. • The identified sets of differentially expressed genes in sea bass scales after exposure to estradiol or genistein provide a source of potential biomarkers for assessing exposure to estrogens, phytoestrogens or other estrogenic pollutants, and this non-invasive approach complies with the 3R principal and is under validation for environmental pollution.

## 1 Data

Table 1 presents the detailed RNA-seq sequence statistics for each of the thirty replicate libraries that were constructed from sea bass scale RNA, grouped according to treatment: fish exposed to estradiol, E2 (E1d and E5d libraries, corresponding to 1 or 5 days of exposure respectively); fish exposed to genistein, Gen (Gen1d and Gen5d) and control fish (C1d and C5d). The transcriptome data released at the NCBI SRA database (Project SRP102504) contains the raw data files of the 30 RNA-seq libraries that were produced from the scales of E2 or Gen-exposed sea bass for each of the six experimental conditions: C1d (experiment with accession SRX2673957, n = 6 replicate libraries), E1d (SRX2673962, n = 5 libraries), Gen1d (SRX2674307, n = 5 libraries), C5d (SRX2674405, n = 5 libraries), E5d (SRX2674467, n = 5 libraries) and Gen5d (SRX2675383, n = 4 libraries).

**Table 1:** Detailed RNA-seq statistics for each library, grouped by treatment. Individual libraries (n = 4–6 from individual fish) were prepared from RNA extracted from scales of fish sampled 1 day after treatment with the vehicle (C1d), estradiol (E1d) or genistein (Gen1d) and scales sampled five days after each treatment (C5d, E5d and Gen5d, respectively). The number of raw and filtered reads (in millions), percentage of mapped reads (for each individual library or on average) and the total numbers of reads produced in the study are presented.

Treatment	Lib name	Raw reads (millions)	Filtered reads (millions)	Mapped reads (%)
C1d	C1d_1	36.2	33.0	89.3%
	C1d_2	32.6	32.6	84.2%
	C1d_3	35.7	35.7	85.6%
	C1d_4	32.0	32.0	85.1%
	C1d_5	32.1	32.1	86.4%
	C1d_6	36.1	36.1	86.2%
E1d	E1d_1	31.5	31.5	87.2%
	E1d_2	37.6	37.6	84.6%
	E1d_3	37.6	37.6	84.8%
	E1d_4	39.2	39.2	84.1%
	E1d_5	31.6	28.3	83.1%
Gen1d	Gen1d_1	41.6	41.6	84.0%
	Gen1d_2	38.5	35.1	85.4%
	Gen1d_3	44.4	44.4	86.1%
	Gen1d_4	30.4	30.4	83.7%
	Gen1d_5	39.2	38.0	84.5%
C5d	C5d_1	40.9	40.9	84.6%
	C5d_2	30.7	30.7	87.3%
	C5d_3	32.1	32.1	86.6%
	C5d_4	29.3	29.3	88.3%
	C5d_5	45.8	45.8	85.6%
E5d	E5d_1	35.4	35.4	85.6%
	E5d_2	36.1	36.1	85.9%
	E5d_3	38.8	26.2	87.0%
	E5d_4	51.0	49.0	88.9%
	E5d_5	44.0	40.8	91.4%
Gen5d	Gen5d_1	44.0	44.0	84.5%
	Gen5d_2	40.9	40.9	86.1%
	Gen5d_3	42.0	42.0	85.9%
	Gen5d_4	44.3	44.3	85.7%
	**Average**	37.7	36.8	
	**Total**	1131.7	1102.8	

**Fig. 1:**
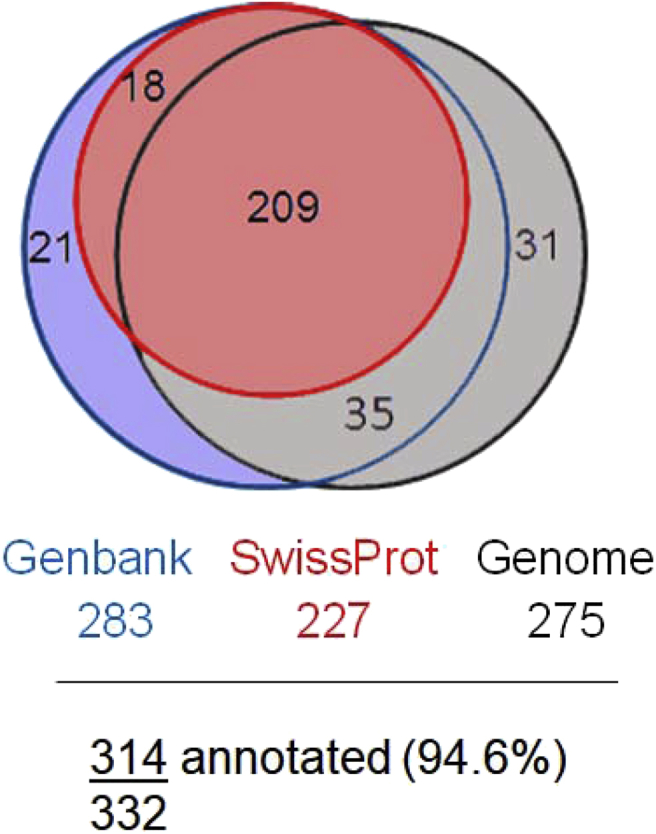
Annotation to different databases of the 332 genes found to be ≥ 2-fold differentially expressed. Venn diagrams indicate the number of genes with a significant match to the sea bass genome, Swiss-Prot protein database or GeneBank protein database, the number of genes annotated by more than one database are inside the intersecting areas. The annotation was carried out in order of preference; 1) matches to Swiss-Prot, 2) matches to the sea bass genome and 3) Genbank matches as indicated by the colour shading. The areas of each sphere are proportional to the number of genes annotated.

**Fig. 2:**
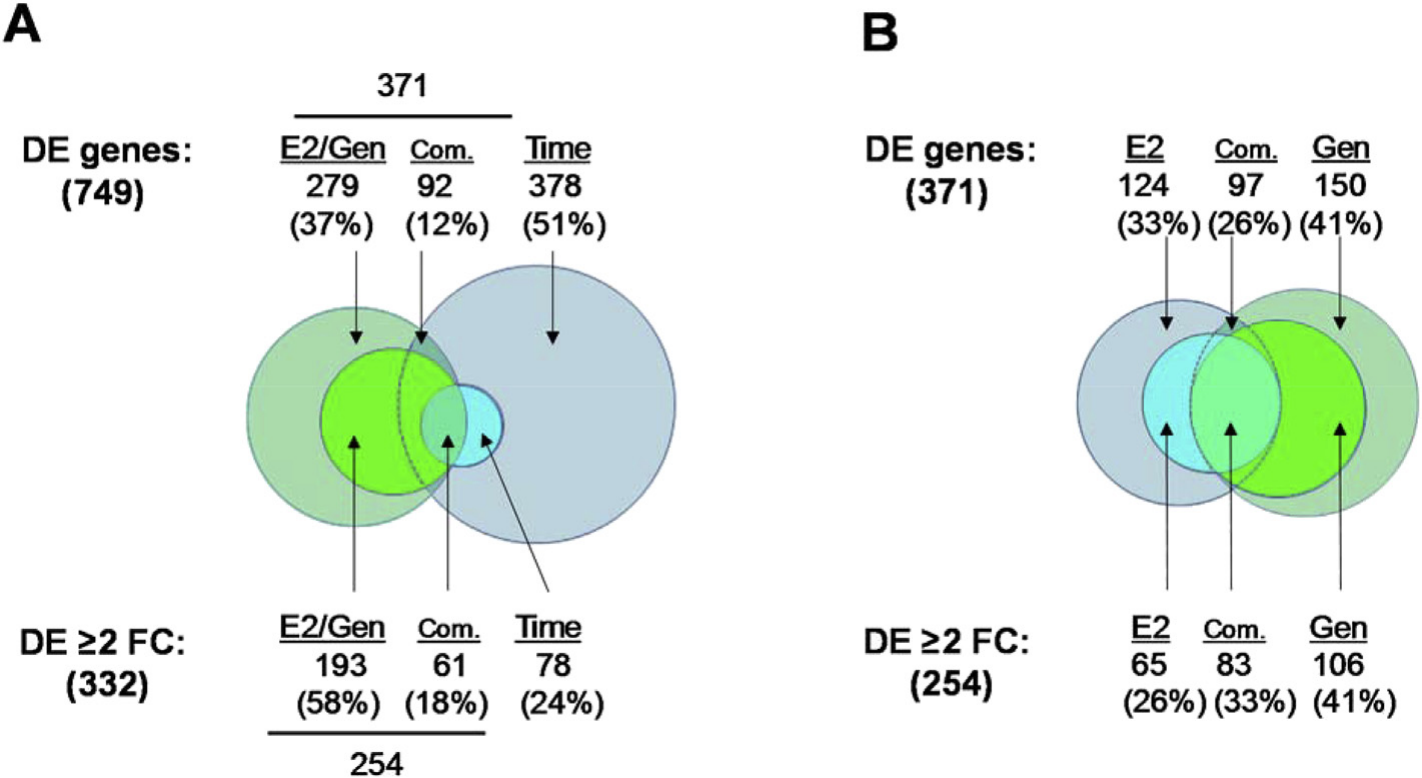
Proportion of differentially expressed (DE) genes identified in the present study. **A**. Venn diagrams representing common (Com.) and specific genes differentially expressed in sea bass scales in response to the treatments (17β-estradiol, E2, and/or genistein, Gen, compared to the corresponding controls at each sampling time 1 day and 5 days), compared to the differential expression in the control groups over time. The number of genes by treatment or time and their respective percentage are shown for two levels of stringency. “DE genes” above the diagram corresponds to the 749 genes differentially expressed at an FDR <0.05. Below the diagram the DE genes (332) differentially expressed with a minimum of 2-fold change are considered (“DE ≥ 2 FC” and FDR < 0.05). 51% of the “DE genes” changed expression only in control scales over time but these changes were of low magnitude (average fold change of 1.9-fold between C1d and C5d) and when the analysis stringency was increased to a minimum of 2-fold change, only 24% of these 332 genes changed expression in the control scales over time. The 254 genes (76%) significantly regulated by E2 or Gen were selected for further analyses. **B**. Shows the comparison of common/specific DE genes between E2 and Gen at the two stringency levels, FDR <0.05 or ≥2 FC and FDR <0.05 (irrespective of the sampling times). For the number of genes regulated by E2 and/or Gen at each sampling time see Fig. 2B of the associated paper in JSBMB [1].

**Fig. 3:**
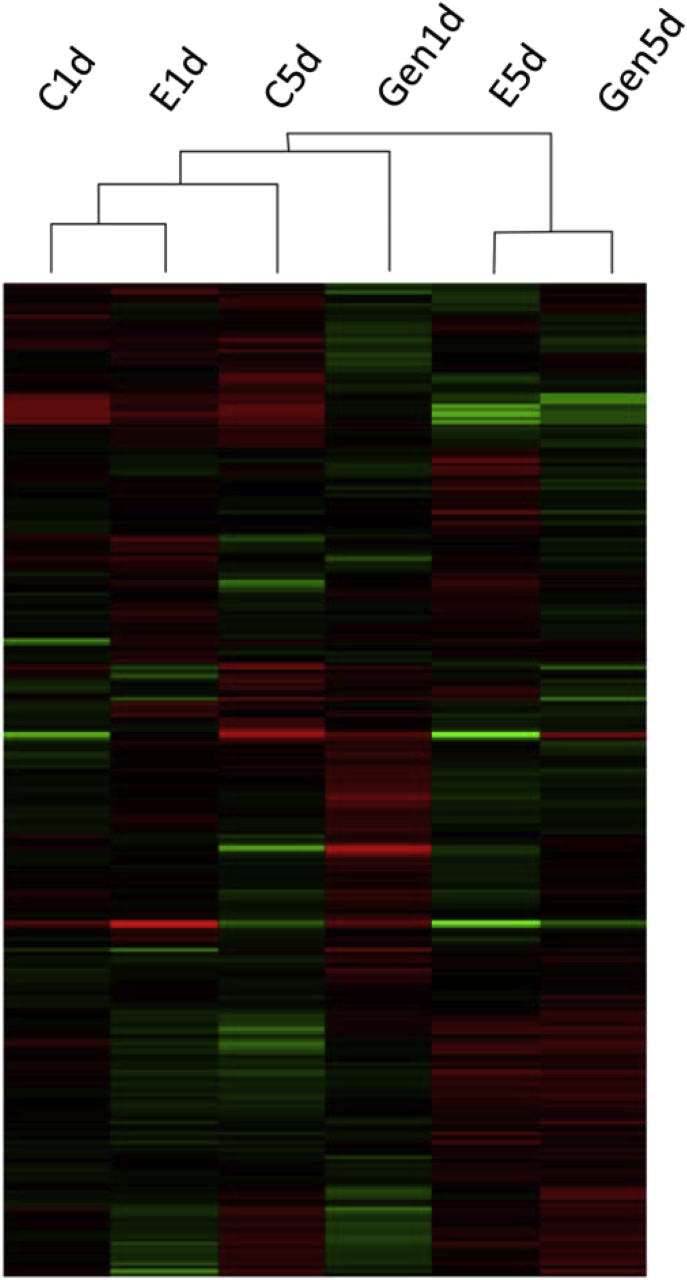
Heatmap of clustered DE genes identified in sea bass scales after treatment with E2 or Gen. The tree in the upper panel shows the hierarchical clustering of the DE genes (one gene/line) identified in the scales of the six treatment groups [injections with estradiol (E), genistein (G) or vehicle only (control, C) at 1 or 5 days (1d or 5d)]. The red gradient indicates high abundance, the green gradient indicates low abundance and black indicates equal abundance for each gene and condition relative to the average. 1 day after treatment the DE genes of the E2 group clustered more closely to the control than Gen1d. 5 days after treatment both E2-and Gen-treated scales clearly separated from the control and clustered together, suggesting a similar response at this time point.

A total of 749 differentially expressed (DE) genes were identified. DE genes in the scale transcriptomes of E2-treated or Gen-treated sea bass were identified by comparison with the corresponding controls at each sampling time and genes changing expression in the control groups when comparing 1 day *vs* 5 days of exposure. The expression levels, fold changes and identities of the 332 DE genes in sea bass scales, that had more than a two-fold change in expression (“DE ≥ 2 FC”), are listed in Supplementary Table 1. A short version of this table (containing only the first 20 genes) is displayed in Table 2. Supplementary Table 2 contains the remaining 417 DE genes (FDR < 0.05 and < 2-fold changes) and an abbreviated version is presented in Table 3. Table 4 presents the detailed results from the annotation of the total list of 749 DE genes (that were used for global enrichment analyses), as well as for the selection of the 332 DE genes with ≥ 2-fold change. Fig. 1 summarizes the preference order strategy used to automatically annotate the 332 selected DE genes using multiple databases, which was followed by careful manual curation. More details about the annotation strategy can be found in the methods below and in the methods and results of the associated JSBMB manuscript [1]. Fig. 2 summarizes the number of genes that were DE in response to the treatments (E2 or Gen) or changed expression between sampling times, when considering different stringency levels: False Discovery Rate (FDR) < 0.05 and < or ≥ 2-fold change in expression. Fig. 3 presents a heatmap showing the grouping of the six treatment groups, according to the identified transcriptome changes in sea bass scales.

**Table 2:** Selected list (first 20 genes) of the genes differentially expressed with a ≥2-fold difference in sea bass scales from treated versus control animals. The complete list with the expression and annotation details for the 332 genes with significant differential expression (FDR < 0.05 and FC ≥ 2) in each comparison can be consulted in Supplementary Table 1. Normalized expression levels in each experimental group are presented in fragments per kilobase of exon model per million reads mapped (fpkm) and significant differential expression for each comparison is presented in Log2 fold change (FC). The final annotation reached using the preference order strategy (Fig. 1 and Table 4) and revised by manual curation is presented, with the accession numbers from Swiss-Prot, Genbank or from the sea bass genome.

Gene ID	Relative expression levels (fpkm)						Log2FC of E2 or Gen effects vs control at 1d				Log2FC of E2 or Gen effects vs control at 5d				Log2FC of C5d vs C1d		Final gene annotation		
Cufflinks#	C1d	E1d	Gen1d	C5d	E5d	Gen5d	up in E2	Down in E2	Up in Gen	Down in Gen	E2 up	E2 down	Gen up	Gen down	C5d up	C5d down	Accession	Hit description	Symbol
XLOC_019555	5.4	14.8	37.7	33.7	5.3	34.0	1.4	–	2.8	–	–	−2.7	–	–	2.6	–	O75443	Alpha-tectorin	TECTA
XLOC_019129	34.2	225.7	761.3	1735.6	22.0	1398.8	2.7	–	4.5	–	–	−6.3	–	–	5.7	–	DLAgn_00248120	Mhc class ii antigen beta chain	HLADPB1
XLOC_020058	0.5	3.8	4.0	3.1	6.2	6.0	2.9	–	2.9	–	–	–	–	–	2.6	–	Q5W7F1	Neutral ceramidase or N-acylsphingosine amidohydrolase 2	ASAH2
XLOC_019225	25.2	63.2	50.8	25.0	17.3	22.9	1.3	–	1.0	–	–	–	–	–	–	–	Q02817	Mucin-2	MUC2
XLOC_019447	10.8	22.6	22.4	12.0	13.6	11.7	1.1	–	1.0	–	–	–	–	–	–	–	XP_019109670.1	Integumentary mucin C.1-like	MUCC1
XLOC_019084	20.6	47.4	38.1	15.9	14.5	40.0	1.2	–	–	–	–	–	1.3	–	–	–	G8HTB6	ZP domain-containing protein / CUB and zona pellucida like domains 1	CUZD1
XLOC_019425	437.4	1926.9	1162.0	1718.5	2043.4	2161.0	2.1	–	–	–	–	–	–	–	–	–	P28064	Proteasome subunit beta type-8	PSMB8
XLOC_021846	92.1	215.6	136.4	206.0	144.3	113.2	1.2	–	–	–	–	–	–	–	1.2	–	–	No hit found	–
XLOC_020866	5.0	19.1	5.9	0.6	0.4	1.1	1.9	–	–	–	–	–	–	–	–	–	DLAgn_00214300	Protein fam111a-like	FAM111A
XLOC_009298	49.5	135.9	60.4	73.6	55.8	70.0	1.5	–	–	–	–	–	–	–	–	–	DLAgn_00110020	Uncharacterized protein loc101483146	hyp_loc101483146
XLOC_020865	260.9	1274.3	269.3	12.9	5.6	19.7	2.0	–	–	–	–	–	–	–	–	–	DLAgn_00214300	Protein fam111a-like	FAM111A
XLOC_018302	1.6	3.9	2.2	1.6	3.5	1.5	1.3	–	–	–	–	–	–	–	–	–	XP_006805317.1	Uncharacterized protein LOC102781223	hyp_LOC102781223
XLOC_001638	3.9	4.2	9.2	3.2	6.8	7.9	–	–	1.2	–	1.1	–	1.3	–	–	–	P35448	Thrombospondin-1	THBS1
XLOC_009985	1.1	0.6	2.8	0.6	1.9	2.9	–	–	1.4	–	–	–	2.2	–	–	–	P43300	Early growth response protein 3	EGR3
XLOC_009459	2.8	2.5	5.8	2.4	4.4	6.1	–	–	1.0	–	–	–	1.4	–	–	–	Q9ET55	Nocturnin	CCRN4L
XLOC_015202	6.1	6.1	19.3	3.5	5.0	8.4	–	–	1.7	–	–	–	1.3	–	–	–	Q16690	Dual specificity protein phosphatase 5	DUSP5
XLOC_009646	8.0	5.6	19.0	3.0	7.6	13.2	–	–	1.2	–	–	–	2.2	–	–	−1.4	Q20A00	DNA damage-inducible transcript 4-like protein	DDIT4L
XLOC_018946	261.3	451.9	567.7	482.9	396.7	190.1	–	–	1.1	–	–	–	–	−1.3	–	–	DLAgn_00241010	Complement c1q-like protein 4 precursor	C1QL4
XLOC_011902	6.6	8.2	13.4	20.8	25.4	9.1	–	–	1.0	–	–	–	–	−1.2	1.7	–	XP_005946242.1	RNA-directed DNA polymerase from mobile element jockey-like	pred_pol413
XLOC_002574	0.8	1.4	2.0	0.8	0.9	1.0	–	–	1.3	–	–	–	–	–	–	–	Q61391	Neprilysin / membrane metallo-endopeptidase	MME

**Table 3:** Selected list (first 20 genes) of the genes differentially expressed at FDR < 0.05 and at < 2-fold change difference, in sea bass scales from treated versus control animals. The complete list with the expression and annotation for the 417 genes with differential expression under these limits (FDR < 0.05 and FC < 2) can be consulted in Supplementary Table 2. Normalized expression levels in each experimental group are presented in fragments per kilobase of exon model per million reads mapped (fpkm) and significant differential expression for each comparison is presented in Log2 fold change (FC). The final annotation reached using the preference order strategy (Fig. 1 and Table 4) is presented, with the accession numbers from Swiss-Prot, Genbank or from the sea bass genome.

Gene ID	Relative expression levels (fpkm)						Log2FC of E2 or Gen effects vs control at 1d				Log2FC of E2 or Gen effects vs control at 5d				Log2FC of C5d vs C1d		Final gene annotation	
Cufflinks#	C1d	E1d	Gen1d	C5d	E5d	Gen5d	E2 up	E2 down	Gen up	Gen down	E2 up	E2 down	Gen up	Gen down	C5d up	C5d down	Hit description	Symbol
XLOC_017886	20.5	32.3	35.2	21.7	28.2	26.6	0.7	–	0.8	–	–	–	–	–	–	–	sp|P51890|LUM_CHICK Lumican OS=Gallus gallus	LUM
XLOC_009066	20.1	32.2	23.6	14.9	18.8	16.6	0.7	–	–	–	–	–	–	–	–	–	sp|Q6NVM0|H10_XENTR Histone H1.0	H1F0
XLOC_000057	39.9	63.9	54.8	51.8	66.1	52.3	0.7	–	–	–	–	–	–	–	–	–	sp|Q04857|CO6A1_MOUSE Collagen alpha-1(VI) chain	COL6A1
XLOC_014484	17.5	28.2	25.8	20.4	14.9	17.2	0.7	–	–	–	–	–	–	–	–	–	sp|Q53RD9|FBLN7_HUMAN Fibulin-7	FBLN7
XLOC_019995	42.4	68.5	41.0	32.9	42.9	22.8	0.7	–	–	–	–	–	–	–	–	–	sp|P55918|MFAP4_BOVIN Microfibril-associated glycoprotein 4	MFAP4
XLOC_006221	7.3	11.8	7.0	7.3	10.5	6.6	0.7	–	–	–	–	–	–	–	–	–	sp|Q4R6P7|SESN1_MACFA Sestrin-1	SESN1
XLOC_002145	58.5	95.2	82.9	108.5	94.2	73.3	0.7	–	–	–	–	–	–	–	0.9	–	sp|O95428|PPN_HUMAN Papilin	PAPLN
XLOC_022309	58.4	96.0	62.0	45.3	81.2	35.5	0.7	–	–	–	0.8	–	–	–	–	–	sp|Q9U8W8|TL5A_TACTR Techylectin-5A	–
XLOC_003581	10.8	18.6	11.2	12.6	8.8	6.9	0.8	–	–	–	–	–	–	−0.9	–	–	sp|Q802Y8|ZB16A_DANRE Zinc finger and BTB domain-containing protein 16-A	ZBTB16
XLOC_011142	1033.0	1793.0	979.6	1063.6	1387.2	709.6	0.8	–	–	–	–	–	–	–	–	–	sp|Q66S03|LECG_THANI Galactose-specific lectin nattectin	LADD
XLOC_013333	40.4	70.4	59.7	50.9	50.7	48.9	0.8	–	–	–	–	–	–	–	–	–	sp|Q90611|MMP2_CHICK 72 kDa type IV collagenase	MMP2
XLOC_020568	323.2	571.3	392.8	275.6	447.7	280.9	0.8	–	–	–	–	–	–	–	–	–	gap junction epsilon-1	–
XLOC_016220	5.0	8.9	6.1	6.5	5.6	5.4	0.8	–	–	–	–	–	–	–	–	–	sp|Q568Y7|NOE2_RAT Noelin-2	OLFM2
XLOC_006639	175.0	315.2	258.5	221.0	268.7	245.3	0.8	–	–	–	–	–	–	–	–	–	sp|Q01584|LIPO_BUFMA Lipocalin	LCN1
XLOC_015159	46.9	88.4	67.0	84.6	64.2	95.1	0.9	–	–	–	–	–	–	–	0.9	–	sp|P27590|UROM_RAT Uromodulin	UMOD
XLOC_019689	26.0	49.4	26.6	17.3	18.0	17.4	0.9	–	–	–	–	–	–	–	–	–	No hit found	–
XLOC_014167	3.2	6.1	5.1	3.4	2.6	2.7	1.0	–	–	–	–	–	–	–	–	–	sp|Q5E9P5|PAMR1_BOVIN Inactive serine protease PAMR1 OS=Bos taur... 413 e-114	PAMR1
XLOC_022211	101.7	197.8	148.2	85.2	76.3	155.6	1.0	–	–	–	–	–	–	–	–	–	No hit found	–
XLOC_008732	3.6	6.9	6.7	5.9	4.9	5.3	1.0	–	0.9	–	–	–	–	–	–	–	sp|Q9BQB4|SOST_HUMAN Sclerostin	SOST
XLOC_018728	132.5	258.4	160.0	121.5	235.3	99.0	1.0	–	–	–	1.0	–	–	–	–	–	No hit found	–

**Table 4:** Detailed results from the annotation. Annotation results (in number of genes or percentage from total) are shown for the 749 genes differentially expressed with q < 0.05 (columns “DE all”) and for the selection of 332 genes differentially expressed at q < 0.05 with a minimum 2-fold change (columns “DE ≥ 2 FC”).

	DE all		DE ≥ 2 FC	
	Number	%	Number	%
**Annotation to different databases:**				
Genes with BlastX hit to SwissProt	593	79	229	68
Genes assigned to predicted genes in genome	600	80	275	83
Genes with BlastX hit to GenBank	676	90	283	85
**Final annotation using preference order:**				
Genes annotated via SwissProt	593	79	229	68
Genes annotated via the sea bass genome	92	12	61	19
Genes annotated via GenBank	34	5	24	7
**Annotated**	719	96	314	95
**Non-annotated**	30	4	18	5
**Total number of DE genes**	749	100	332	100
**Summary of annotation:**				
Annotation to known proteins	667	89	280	84
Annotation to predicted proteins	21	3	12	4
Annotation to hypothetical/uncharacterized proteins	20	3	14	4
Mapping to non-annotated genes	11	1	8	2

Table 5, Table 6 present the significantly enriched GO Biological Processes (GO-BP) and KEGG pathways, respectively, of all genes that presented significant changes in expression in response to the treatments E2 and/or Gen (analysis “All”); Table 7, Table 8 present the significantly enriched GO-BP and KEGGs when directly comparing the E2-or Gen-responsive genes, irrespective of the sampling time (analyses “E2” vs “Gen”); Table 9, Table 10 list the significantly enriched GO-BP and KEGGs when comparing responsive gene lists between 1 day and 5 days (analyses “1d” vs “5d”).

**Table 5:** Enrichment of GO Biological Processes (GO-BP) using all genes found to be differentially expressed in response to E2 and/or Gen (analysis “All”). Significantly enriched biological processes (FDR < 0.05) were identified by ID and term description and grouped into 23 functionally related networks (GO group) obtained by ClueGO analysis. Each group is named after its most significant term (lowest FDR) and highlighted in bold, which was chosen for GOTerm representation in Fig. 3 of the associated MS in JSBMB [1]. Functionally related groups are sorted by highest enrichment score, calculated as [ -Log2 (group FDR)].

GOID	GOTerm	Term FDR	Group FDR	Enrichment Score	GO group	% Associated Genes	Nr. Genes
GO:0043207	**response to external biotic stimulus**	7.2E-10	1.8E-10	32.4	18	8.54	21.00
GO:0051707	response to other organism	7.2E-10	1.8E-10	32.4	18	8.54	21.00
GO:0009615	response to virus	3.8E-05	1.8E-10	32.4	18	12.96	7.00
GO:0009617	response to bacterium	1.5E-03	1.8E-10	32.4	18	5.20	9.00
GO:0051607	defense response to virus	2.1E-02	1.8E-10	32.4	18	7.14	3.00
GO:0043207	**response to external biotic stimulus**	7.2E-10	5.9E-09	27.3	20	8.54	21.00
GO:0051707	response to other organism	7.2E-10	5.9E-09	27.3	20	8.54	21.00
GO:0051591	response to cAMP	4.8E-05	5.9E-09	27.3	20	75.00	3.00
GO:0009617	response to bacterium	1.5E-03	5.9E-09	27.3	20	5.20	9.00
GO:0046683	response to organophosphorus	2.3E-03	5.9E-09	27.3	20	20.00	3.00
GO:0014074	response to purine-containing compound	2.3E-03	5.9E-09	27.3	20	20.00	3.00
GO:0032496	response to lipopolysaccharide	2.7E-03	5.9E-09	27.3	20	8.33	5.00
GO:0002237	response to molecule of bacterial origin	3.1E-03	5.9E-09	27.3	20	7.94	5.00
GO:0034097	response to cytokine	3.5E-03	5.9E-09	27.3	20	4.29	9.00
GO:0006954	inflammatory response	8.2E-03	5.9E-09	27.3	20	4.32	7.00
GO:0042493	response to drug	1.2E-02	5.9E-09	27.3	20	9.38	3.00
GO:0009612	response to mechanical stimulus	2.5E-02	5.9E-09	27.3	20	6.67	3.00
GO:0016126	**sterol biosynthetic process**	2.9E-09	3.0E-07	21.7	22	31.03	9.00
GO:0006694	steroid biosynthetic process	4.0E-09	3.0E-07	21.7	22	19.30	11.00
GO:0016125	sterol metabolic process	9.6E-09	3.0E-07	21.7	22	20.83	10.00
GO:0008202	steroid metabolic process	1.0E-08	3.0E-07	21.7	22	14.63	12.00
GO:1901617	organic hydroxy compound biosynthetic process	8.7E-07	3.0E-07	21.7	22	15.25	9.00
GO:0008203	cholesterol metabolic process	2.6E-06	3.0E-07	21.7	22	20.59	7.00
GO:1902652	secondary alcohol metabolic process	4.3E-06	3.0E-07	21.7	22	18.92	7.00
GO:0006695	cholesterol biosynthetic process	1.1E-05	3.0E-07	21.7	22	31.25	5.00
GO:1902653	secondary alcohol biosynthetic process	1.8E-05	3.0E-07	21.7	22	27.78	5.00
GO:1901615	organic hydroxy compound metabolic process	4.4E-05	3.0E-07	21.7	22	7.69	10.00
GO:0044283	small molecule biosynthetic process	8.0E-05	3.0E-07	21.7	22	5.85	12.00
GO:0046165	alcohol biosynthetic process	1.3E-04	3.0E-07	21.7	22	17.86	5.00
GO:0008610	lipid biosynthetic process	4.1E-04	3.0E-07	21.7	22	4.86	12.00
GO:0006066	alcohol metabolic process	4.5E-04	3.0E-07	21.7	22	8.33	7.00
GO:0016053	organic acid biosynthetic process	1.3E-03	3.0E-07	21.7	22	5.97	8.00
GO:0046394	carboxylic acid biosynthetic process	1.3E-03	3.0E-07	21.7	22	5.97	8.00
GO:1901607	alpha-amino acid biosynthetic process	1.4E-03	3.0E-07	21.7	22	10.42	5.00
GO:0008652	cellular amino acid biosynthetic process	1.6E-03	3.0E-07	21.7	22	9.80	5.00
GO:0031099	regeneration	3.0E-03	3.0E-07	21.7	22	4.94	8.00
GO:0031329	regulation of cellular catabolic process	3.0E-03	3.0E-07	21.7	22	5.51	7.00
GO:0009894	regulation of catabolic process	3.5E-03	3.0E-07	21.7	22	5.22	7.00
GO:0006520	cellular amino acid metabolic process	6.5E-03	3.0E-07	21.7	22	4.15	8.00
GO:0022600	digestive system process	6.9E-03	3.0E-07	21.7	22	12.00	3.00
GO:1901605	alpha-amino acid metabolic process	7.2E-03	3.0E-07	21.7	22	5.08	6.00
GO:0007586	digestion	1.2E-02	3.0E-07	21.7	22	9.38	3.00
GO:0072330	monocarboxylic acid biosynthetic process	1.5E-02	3.0E-07	21.7	22	5.97	4.00
GO:0031331	positive regulation of cellular catabolic process	1.7E-02	3.0E-07	21.7	22	5.71	4.00
GO:0009896	positive regulation of catabolic process	1.9E-02	3.0E-07	21.7	22	5.48	4.00
GO:0097164	ammonium ion metabolic process	2.9E-02	3.0E-07	21.7	22	6.25	3.00
GO:0015748	organophosphate ester transport	3.5E-02	3.0E-07	21.7	22	5.77	3.00
GO:0006633	fatty acid biosynthetic process	3.5E-02	3.0E-07	21.7	22	5.77	3.00
GO:0001878	**response to yeast**	4.6E-06	8.8E-06	16.8	12	25.00	6.00
GO:0009620	response to fungus	1.2E-05	8.8E-06	16.8	12	20.69	6.00
GO:0031099	**regeneration**	3.0E-03	2.7E-04	11.9	19	4.94	8.00
GO:0009611	response to wounding	3.3E-03	2.7E-04	11.9	19	4.33	9.00
GO:0042060	wound healing	3.4E-03	2.7E-04	11.9	19	4.68	8.00
GO:0022600	digestive system process	6.9E-03	2.7E-04	11.9	19	12.00	3.00
GO:0007586	digestion	1.2E-02	2.7E-04	11.9	19	9.38	3.00
GO:0042246	tissue regeneration	4.3E-02	2.7E-04	11.9	19	4.08	4.00
GO:0007599	hemostasis	4.5E-02	2.7E-04	11.9	19	5.08	3.00
GO:0007596	blood coagulation	4.5E-02	2.7E-04	11.9	19	5.08	3.00
GO:0050817	coagulation	4.9E-02	2.7E-04	11.9	19	4.84	3.00
GO:0030162	**regulation of proteolysis**	3.4E-03	5.6E-03	7.5	11	4.02	10.00
GO:0045861	negative regulation of proteolysis	1.7E-02	5.6E-03	7.5	11	4.03	6.00
GO:0006270	**DNA replication initiation**	6.9E-03	1.2E-02	6.4	0	12.00	3.00
GO:0048593	**camera-type eye morphogenesis**	6.4E-03	1.4E-02	6.2	17	4.64	7.00
GO:0048592	eye morphogenesis	7.3E-03	1.4E-02	6.2	17	4.04	8.00
GO:0042462	eye photoreceptor cell development	1.9E-02	1.4E-02	6.2	17	7.50	3.00
GO:0031076	embryonic camera-type eye development	3.1E-02	1.4E-02	6.2	17	6.12	3.00
GO:0001754	eye photoreceptor cell differentiation	4.8E-02	1.4E-02	6.2	17	4.92	3.00
GO:0006979	**response to oxidative stress**	3.7E-03	1.4E-02	6.2	16	7.25	5.00
GO:1990748	cellular detoxification	3.1E-02	1.4E-02	6.2	16	6.12	3.00
GO:0098869	cellular oxidant detoxification	3.1E-02	1.4E-02	6.2	16	6.12	3.00
GO:0098754	detoxification	3.5E-02	1.4E-02	6.2	16	5.77	3.00
GO:0031589	**cell-substrate adhesion**	9.6E-03	1.5E-02	6.1	10	7.02	4.00
GO:0007160	cell-matrix adhesion	3.2E-02	1.5E-02	6.1	10	6.00	3.00
GO:0006730	**one-carbon metabolic process**	1.4E-02	1.8E-02	5.8	4	8.57	3.00
GO:0051241	**negative regulation of multicellular organismal process**	1.3E-02	1.8E-02	5.8	3	4.32	6.00
GO:0009266	**response to temperature stimulus**	1.7E-02	2.1E-02	5.6	8	7.89	3.00
GO:0072527	**pyrimidine-containing compound metabolic process**	1.9E-02	2.3E-02	5.4	5	7.50	3.00
GO:1901136	**carbohydrate derivative catabolic process**	3.2E-02	3.9E-02	4.7	6	6.00	3.00
GO:0009410	**response to xenobiotic stimulus**	3.5E-02	4.0E-02	4.6	9	5.77	3.00
GO:0001945	**lymph vessel development**	3.8E-02	4.2E-02	4.6	2	5.56	3.00
GO:0048864	**stem cell development**	4.1E-02	4.3E-02	4.5	13	4.17	4.00
GO:0014031	mesenchymal cell development	4.1E-02	4.3E-02	4.5	13	4.17	4.00
GO:0014032	neural crest cell development	4.1E-02	4.3E-02	4.5	13	4.17	4.00
GO:0000188	**inactivation of MAPK activity**	3.4E-04	4.6E-02	4.4	21	22.22	4.00
GO:0043407	negative regulation of MAP kinase activity	5.6E-04	4.6E-02	4.4	21	19.05	4.00
GO:0043409	negative regulation of MAPK cascade	1.4E-03	4.6E-02	4.4	21	14.29	4.00
GO:0043405	regulation of MAP kinase activity	2.0E-03	4.6E-02	4.4	21	7.32	6.00
GO:0071901	negative regulation of protein serine/threonine kinase activity	2.1E-03	4.6E-02	4.4	21	12.50	4.00
GO:0033673	negative regulation of kinase activity	2.7E-03	4.6E-02	4.4	21	6.67	6.00
GO:0006469	negative regulation of protein kinase activity	2.7E-03	4.6E-02	4.4	21	6.74	6.00
GO:0051348	negative regulation of transferase activity	3.3E-03	4.6E-02	4.4	21	6.25	6.00
GO:0001933	negative regulation of protein phosphorylation	3.4E-03	4.6E-02	4.4	21	6.12	6.00
GO:0042326	negative regulation of phosphorylation	3.6E-03	4.6E-02	4.4	21	6.00	6.00
GO:1902532	negative regulation of intracellular signal transduction	8.0E-03	4.6E-02	4.4	21	4.96	6.00
GO:0071900	regulation of protein serine/threonine kinase activity	8.2E-03	4.6E-02	4.4	21	4.92	6.00
GO:0010563	negative regulation of phosphorus metabolic process	9.6E-03	4.6E-02	4.4	21	4.72	6.00
GO:0045936	negative regulation of phosphate metabolic process	9.6E-03	4.6E-02	4.4	21	4.72	6.00
GO:0031400	negative regulation of protein modification process	9.6E-03	4.6E-02	4.4	21	4.72	6.00
GO:0001706	endoderm formation	1.6E-02	4.6E-02	4.4	21	8.11	3.00
GO:0001704	formation of primary germ layer	1.9E-02	4.6E-02	4.4	21	5.48	4.00
GO:0001666	**response to hypoxia**	4.4E-02	4.6E-02	4.4	15	5.17	3.00
GO:0070482	response to oxygen levels	4.6E-02	4.6E-02	4.4	15	5.00	3.00
GO:0036293	response to decreased oxygen levels	4.6E-02	4.6E-02	4.4	15	5.00	3.00
GO:0009142	**nucleoside triphosphate biosynthetic process**	4.5E-02	4.7E-02	4.4	7	5.08	3.00
GO:0033334	**fin morphogenesis**	4.5E-02	4.7E-02	4.4	1	5.08	3.00
GO:0042541	**hemoglobin biosynthetic process**	1.7E-03	6.2E-02	4.0	14	23.08	3.00
GO:0020027	hemoglobin metabolic process	2.0E-03	6.2E-02	4.0	14	21.43	3.00
GO:0055076	transition metal ion homeostasis	4.9E-02	6.2E-02	4.0	14	4.84	3.00

**Table 6:** Enrichment of KEGG pathways using all genes found to be differentially expressed in response to E2 and/or Gen (analysis “All”). Significantly enriched pathways (FDR < 0.05) were identified by their KEGG identifier and have been grouped according to 7 functionally related groups obtained by ClueGO analysis. Each group is named after its most significant term (lowest FDR), which is indicated in bold. Functionally related groups are sorted by highest enrichment score, calculated as [- Log2 (group FDR)].

KEGG Identifier	KEGG ID FDR	Group FDR	Enrichment Score	Groups	% Associated Genes	Nr. Genes
Steroid biosynthesis	240.0E-9	210.0E-9	22.2	0	33.33	7.00
ECM-receptor interaction	1.5E-3	1.3E-3	9.6	5	8.64	7.00
Alanine, aspartate and glutamate metabolism	8.7E-3	3.0E-3	8.4	6	10.00	4.00
Arginine biosynthesis	10.0E-3	3.0E-3	8.4	6	12.00	3.00
DNA replication	9.6E-3	6.3E-3	7.3	2	10.53	4.00
p53 signaling pathway	12.0E-3	11.0E-3	6.5	4	6.76	5.00
Arachidonic acid metabolism	11.0E-3	12.0E-3	6.4	1	7.84	4.00
Proteasome	14.0E-3	14.0E-3	6.2	3	7.14	4.00

**Table 7:** Enrichment of GO Biological Processes (GO-BP) by the genes differentially expressed in response to E2 or to Gen (analyses “E2” vs “Gen”). Significantly enriched biological processes (FDR < 0.05), identified by their ID and term description, are grouped into 12 functionally related networks (GO group) obtained by ClueGO analysis. Each group is named after its most significant term (lowest FDR), highlighted in bold, which was chosen for group representation in Fig. 3 of the associated MS in JSBMB [1]. Functionally related groups are sorted by the highest enrichment score, calculated as [- Log2 (group FDR)]. The classification of each term as specifically enriched in response to the E2 or Gen treatments (or both), obtained by ClueGO cluster analysis, is also shown. For groups including terms enriched in more than one treatment, the classification of the leading term was adopted for the group.

GOID	GOTerm	Term FDR	Group FDR	Enrichment Score	GOGroups	% Associated Genes	Nr. Genes	Treatment	%Genes Specific for E2	%Genes Specific for Gen
GO:0043207	**Response to external biotic stimulus**	540.0E-12	100.0E-12	33.2	7	8.54	21.00	Specific for Gen	51.67	73.19
GO:0051707	response to other organism	540.0E-12	100.0E-12	33.2	7	8.54	21.00	Specific for Gen	51.67	73.19
GO:0009615	response to virus	28.0E-6	100.0E-12	33.2	7	12.96	7.00	Specific for Gen	50.61	63.26
GO:0009617	response to bacterium	1.1E-3	100.0E-12	33.2	7	5.20	9.00	Specific for Gen	55.51	74.01
GO:0043207	**Response to external biotic stimulus**	540.0E-12	8.9E-9	26.7	9	8.54	21.00	Specific for Gen	51.67	73.19
GO:0051707	response to other organism	540.0E-12	8.9E-9	26.7	9	8.54	21.00	Specific for Gen	51.67	73.19
GO:0051591	response to cAMP	36.0E-6	8.9E-9	26.7	9	75.00	3.00	Specific for Gen	45.51	68.26
GO:0009617	response to bacterium	1.1E-3	8.9E-9	26.7	9	5.20	9.00	Specific for Gen	55.51	74.01
GO:0046683	response to organophosphorus	1.8E-3	8.9E-9	26.7	9	20.00	3.00	Specific for Gen	45.51	68.26
GO:0014074	response to purine-containing compound	1.8E-3	8.9E-9	26.7	9	20.00	3.00	Specific for Gen	45.51	68.26
GO:0032496	response to lipopolysaccharide	2.0E-3	8.9E-9	26.7	9	8.33	5.00	Specific for Gen	49.63	66.17
GO:0002237	response to molecule of bacterial origin	2.3E-3	8.9E-9	26.7	9	7.94	5.00	Specific for Gen	49.63	66.17
GO:0042493	response to drug	10.0E-3	8.9E-9	26.7	9	9.38	3.00	Specific for Gen	45.51	68.26
GO:0009612	response to mechanical stimulus	23.0E-3	8.9E-9	26.7	9	6.67	3.00	Specific for Gen	45.51	68.26
GO:0016126	**Sterol biosynthetic process**	2.2E-9	1.3E-6	19.6	11	31.03	9.00	Specific for Gen	20.36	91.63
GO:0006694	steroid biosynthetic process	3.0E-9	1.3E-6	19.6	11	19.30	11.00	Specific for Gen	17.00	93.49
GO:0016125	sterol metabolic process	7.3E-9	1.3E-6	19.6	11	20.83	10.00	Specific for Gen	18.53	92.66
GO:0008202	steroid metabolic process	7.7E-9	1.3E-6	19.6	11	14.63	12.00	Specific for Gen	15.69	94.16
GO:1901617	organic hydroxy compound biosynthetic process	660.0E-9	1.3E-6	19.6	11	15.25	9.00	Specific for Gen	20.36	91.63
GO:0008203	cholesterol metabolic process	2.0E-6	1.3E-6	19.6	11	20.59	7.00	Specific for Gen	25.30	88.56
GO:1902652	secondary alcohol metabolic process	3.2E-6	1.3E-6	19.6	11	18.92	7.00	Specific for Gen	25.30	88.56
GO:0006695	cholesterol biosynthetic process	8.7E-6	1.3E-6	19.6	11	31.25	5.00	Specific for Gen	17.96	89.82
GO:1902653	secondary alcohol biosynthetic process	13.0E-6	1.3E-6	19.6	11	27.78	5.00	Specific for Gen	17.96	89.82
GO:1901615	organic hydroxy compound metabolic process	33.0E-6	1.3E-6	19.6	11	7.69	10.00	Specific for Gen	18.53	92.66
GO:0044283	small molecule biosynthetic process	60.0E-6	1.3E-6	19.6	11	5.85	12.00	Specific for Gen	29.87	89.62
GO:0046165	alcohol biosynthetic process	100.0E-6	1.3E-6	19.6	11	17.86	5.00	Specific for Gen	17.96	89.82
GO:0008610	lipid biosynthetic process	310.0E-6	1.3E-6	19.6	11	4.86	12.00	Specific for Gen	15.69	94.16
GO:0006066	alcohol metabolic process	340.0E-6	1.3E-6	19.6	11	8.33	7.00	Specific for Gen	25.30	88.56
GO:0016053	organic acid biosynthetic process	1.0E-3	1.3E-6	19.6	11	5.97	8.00	Specific for Gen	32.52	86.72
GO:0046394	carboxylic acid biosynthetic process	1.0E-3	1.3E-6	19.6	11	5.97	8.00	Specific for Gen	32.52	86.72
GO:1901607	alpha-amino acid biosynthetic process	1.0E-3	1.3E-6	19.6	11	10.42	5.00	Specific for Gen	33.08	82.71
GO:0008652	cellular amino acid biosynthetic process	1.2E-3	1.3E-6	19.6	11	9.80	5.00	Specific for Gen	33.08	82.71
GO:0031099	Regeneration	2.2E-3	1.3E-6	19.6	11	4.94	8.00	Specific for Gen	32.52	86.72
GO:0006520	cellular amino acid metabolic process	5.6E-3	1.3E-6	19.6	11	4.15	8.00	Specific for Gen	22.58	90.31
GO:0022600	digestive system process	6.0E-3	1.3E-6	19.6	11	12.00	3.00	Specific for Gen	26.42	79.25
GO:1901605	alpha-amino acid metabolic process	6.3E-3	1.3E-6	19.6	11	5.08	6.00	Specific for Gen	28.72	86.17
GO:0007586	Digestion	10.0E-3	1.3E-6	19.6	11	9.38	3.00	Specific for Gen	26.42	79.25
GO:0072330	monocarboxylic acid biosynthetic process	14.0E-3	1.3E-6	19.6	11	5.97	4.00	Specific for Gen	38.69	77.37
GO:0031331	positive regulation of cellular catabolic process	15.0E-3	1.3E-6	19.6	11	5.71	4.00	Both treatments	58.03	58.03
GO:0009896	positive regulation of catabolic process	17.0E-3	1.3E-6	19.6	11	5.48	4.00	Both treatments	58.03	58.03
GO:0097164	ammonium ion metabolic process	27.0E-3	1.3E-6	19.6	11	6.25	3.00	Specific for Gen	26.42	79.25
GO:0015748	organophosphate ester transport	33.0E-3	1.3E-6	19.6	11	5.77	3.00	Specific for Gen	26.42	79.25
GO:0006633	fatty acid biosynthetic process	33.0E-3	1.3E-6	19.6	11	5.77	3.00	Specific for Gen	26.42	79.25
GO:0001878	**Response to yeast**	3.4E-6	4.8E-6	17.7	4	25.00	6.00	Specific for Gen	43.08	71.80
GO:0009620	response to fungus	9.5E-6	4.8E-6	17.7	4	20.69	6.00	Specific for Gen	43.08	71.80
GO:0031099	**Regeneration**	2.2E-3	350.0E-6	11.5	8	4.94	8.00	Specific for Gen	32.52	86.72
GO:0042060	wound healing	2.8E-3	350.0E-6	11.5	8	4.68	8.00	Specific for Gen	52.30	73.22
GO:0022600	digestive system process	6.0E-3	350.0E-6	11.5	8	12.00	3.00	Specific for Gen	26.42	79.25
GO:0007586	Digestion	10.0E-3	350.0E-6	11.5	8	9.38	3.00	Specific for Gen	26.42	79.25
GO:0042246	tissue regeneration	41.0E-3	350.0E-6	11.5	8	4.08	4.00	Specific for Gen	38.69	77.37
GO:0007599	Hemostasis	43.0E-3	350.0E-6	11.5	8	5.08	3.00	Specific for E2	68.26	45.51
GO:0007596	blood coagulation	43.0E-3	350.0E-6	11.5	8	5.08	3.00	Specific for E2	68.26	45.51
GO:0050817	Coagulation	48.0E-3	350.0E-6	11.5	8	4.84	3.00	Specific for E2	68.26	45.51
GO:0006979	**Response to oxidative stress**	3.1E-3	3.9E-3	8.0	1	7.25	5.00	**Both treatments**	53.89	53.89
GO:0031589	**cell-substrate adhesion**	8.6E-3	10.0E-3	6.6	5	7.02	4.00	Specific for E2	64.60	43.07
GO:0007160	cell-matrix adhesion	30.0E-3	10.0E-3	6.6	5	6.00	3.00	Specific for E2	79.25	26.42
GO:0006730	**One-carbon metabolic process**	13.0E-3	13.0E-3	6.3	0	8.57	3.00	Specific for Gen	0.00	100.00
GO:0051241	negative regulation of multicellular organismal process	12.0E-3	14.0E-3	6.2	3	4.32	6.00	Specific for Gen	51.88	77.82
GO:0001945	**Lymph vessel development**	35.0E-3	35.0E-3	4.8	2	5.56	3.00	Specific for E2	68.26	45.51
GO:0000188	**Inactivation of MAPK activity**	260.0E-6	39.0E-3	4.7	10	22.22	4.00	Specific for Gen	53.43	71.24
GO:0043407	negative regulation of MAP kinase activity	420.0E-6	39.0E-3	4.7	10	19.05	4.00	Specific for Gen	53.43	71.24
GO:0043409	negative regulation of MAPK cascade	1.1E-3	39.0E-3	4.7	10	14.29	4.00	Specific for Gen	53.43	71.24
GO:0043405	regulation of MAP kinase activity	1.5E-3	39.0E-3	4.7	10	7.32	6.00	Both treatments	64.85	64.85
GO:0071901	negative regulation of protein serine/threonine kinase activity	1.5E-3	39.0E-3	4.7	10	12.50	4.00	Specific for Gen	53.43	71.24
GO:0033673	negative regulation of kinase activity	2.1E-3	39.0E-3	4.7	10	6.67	6.00	Both treatments	64.85	64.85
GO:0006469	negative regulation of protein kinase activity	2.1E-3	39.0E-3	4.7	10	6.74	6.00	Both treatments	64.85	64.85
GO:0051348	negative regulation of transferase activity	2.6E-3	39.0E-3	4.7	10	6.25	6.00	Both treatments	64.85	64.85
GO:0001933	negative regulation of protein phosphorylation	2.9E-3	39.0E-3	4.7	10	6.12	6.00	Both treatments	64.85	64.85
GO:0042326	negative regulation of phosphorylation	3.0E-3	39.0E-3	4.7	10	6.00	6.00	Both treatments	64.85	64.85
GO:1902532	negative regulation of intracellular signal transduction	7.0E-3	39.0E-3	4.7	10	4.96	6.00	Both treatments	64.85	64.85
GO:0071900	regulation of protein serine/threonine kinase activity	7.1E-3	39.0E-3	4.7	10	4.92	6.00	Both treatments	64.85	64.85
GO:0001706	endoderm formation	14.0E-3	39.0E-3	4.7	10	8.11	3.00	Specific for Gen	45.51	68.26
GO:0001704	formation of primary germ layer	17.0E-3	39.0E-3	4.7	10	5.48	4.00	Specific for Gen	53.43	71.24
GO:0007492	endoderm development	49.0E-3	39.0E-3	4.7	10	4.76	3.00	Specific for Gen	45.51	68.26
GO:0042541	**Hemoglobin biosynthetic process**	1.3E-3	62.0E-3	4.0	6	23.08	3.00	Specific for Gen	26.42	79.25
GO:0020027	hemoglobin metabolic process	1.5E-3	62.0E-3	4.0	6	21.43	3.00	Specific for Gen	26.42	79.25
GO:0055076	transition metal ion homeostasis	48.0E-3	62.0E-3	4.0	6	4.84	3.00	Specific for Gen	26.42	79.25

**Table 8:** Enrichment of KEGG pathways by the genes differentially expressed in response to E2 or to Gen (Analyses “E2” vs “Gen”). Significantly enriched pathways (FDR < 0.05), identified by their KEGG identifier, are grouped according to 6 functionally related groups obtained by ClueGO analysis. Each group is named after its most significant term (lowest FDR) and is highlighted in bold. Functionally related groups are sorted by highest enrichment score, calculated as [- Log2 (group FDR)].

KEGG Identifier	KEGG ID FDR	Group FDR	Enrichment Score	Groups	% Associated Genes	Nr. Genes	Treatment	%Genes Specific for E2	%Genes Specific for Gen
Steroid biosynthesis	210.0E-9	180.0E-9	22.4	0	33.33	7.00	Specific for Gen	0.00	100.00
ECM-receptor interaction	1.3E-3	1.1E-3	9.8	4	8.64	7.00	Specific for E2	66.84	40.11
Alanine, aspartate and glutamate metabolism	7.6E-3	2.6E-3	8.6	5	10.00	4.00	Specific for Gen	21.53	86.14
Arginine biosynthesis	9.5E-3	2.6E-3	8.6	5	12.00	3.00	Specific for Gen	0.00	100.00
DNA replication	8.4E-3	5.4E-3	7.5	1	10.53	4.00	Specific for Gen	25.00	75.00
p53 signaling pathway	11.0E-3	9.5E-3	6.7	3	6.76	5.00	Both treatments	53.89	53.89
Proteasome	14.0E-3	14.0E-3	6.2	2	7.14	4.00	Specific for E2	86.14	21.53

**Table 9:** Enrichment of GO Biological Processes by the genes differentially expressed after 1 day or 5 days (Analyses “1d” vs “5d”). Significantly enriched biological processes (FDR < 0.05), identified by their ID and term description, are grouped according to 11 functionally related networks (GO group) obtained by ClueGO analysis. Each group is named after its most significant term (lowest FDR), highlighted in bold, which was chosen for GOTerm representation in Fig. 3 of the associated MS in JSBMB [1]. Functionally related groups are sorted by highest enrichment score, calculated as [- Log2 (group FDR)]. The classification of each term as specifically enriched in the responses after 1 day or 5 days (or both), obtained by ClueGO cluster analysis, is also shown. For groups including terms enriched in more than one list the classification of the leading term was adopted for the whole group.

GOID	GOTerm	Term FDR	Group FDR	Enrichment Score	GOGroups	% Associated Genes	Nr. Genes	Time	%Genes Specific for 1d	%Genes Specific for 5d
GO:0043207	**Response to external biotic stimulus**	490.0E-12	100.0E-12	33.2	7	8.54	21.00	Specific for 5 days	36.49	72.99
GO:0051707	response to other organism	490.0E-12	100.0E-12	33.2	7	8.54	21.00	Specific for 5 days	36.49	72.99
GO:0009615	response to virus	26.0E-6	100.0E-12	33.2	7	12.96	7.00	Specific for 5 days	14.29	85.71
GO:0009617	response to bacterium	1.0E-3	100.0E-12	33.2	7	5.20	9.00	Specific for 5 days	39.30	78.60
GO:0043207	**Response to external biotic stimulus**	490.0E-12	8.9E-9	26.7	9	8.54	21.00	Specific for 5 days	36.49	72.99
GO:0051707	response to other organism	490.0E-12	8.9E-9	26.7	9	8.54	21.00	Specific for 5 days	36.49	72.99
GO:0051591	response to cAMP	33.0E-6	8.9E-9	26.7	9	75.00	3.00	Specific for 5 days	45.51	68.26
GO:0009617	response to bacterium	1.0E-3	8.9E-9	26.7	9	5.20	9.00	Specific for 5 days	39.30	78.60
GO:0046683	response to organophosphorus	1.7E-3	8.9E-9	26.7	9	20.00	3.00	Specific for 5 days	45.51	68.26
GO:0014074	response to purine-containing compound	1.7E-3	8.9E-9	26.7	9	20.00	3.00	Specific for 5 days	45.51	68.26
GO:0032496	response to lipopolysaccharide	1.9E-3	8.9E-9	26.7	9	8.33	5.00	Specific for 5 days	33.08	82.71
GO:0002237	response to molecule of bacterial origin	2.2E-3	8.9E-9	26.7	9	7.94	5.00	Specific for 5 days	33.08	82.71
GO:0042493	response to drug	10.0E-3	8.9E-9	26.7	9	9.38	3.00	Specific for 5 days	45.51	68.26
GO:0009612	response to mechanical stimulus	23.0E-3	8.9E-9	26.7	9	6.67	3.00	Specific for 5 days	45.51	68.26
GO:0016126	**Sterol biosynthetic process**	2.0E-9	76.0E-9	23.6	11	31.03	9.00	Specific for 1 day	84.82	21.21
GO:0006694	steroid biosynthetic process	2.8E-9	76.0E-9	23.6	11	19.30	11.00	Specific for 1 day	78.95	26.32
GO:0016125	sterol metabolic process	6.6E-9	76.0E-9	23.6	11	20.83	10.00	Specific for 1 day	86.42	19.21
GO:0008202	steroid metabolic process	7.0E-9	76.0E-9	23.6	11	14.63	12.00	Specific for 1 day	80.73	24.22
GO:1901617	organic hydroxy compound biosynthetic process	600.0E-9	76.0E-9	23.6	11	15.25	9.00	Specific for 1 day	84.82	21.21
GO:0008203	cholesterol metabolic process	1.8E-6	76.0E-9	23.6	11	20.59	7.00	Specific for 1 day	80.21	26.74
GO:1902652	secondary alcohol metabolic process	2.9E-6	76.0E-9	23.6	11	18.92	7.00	Specific for 1 day	80.21	26.74
GO:0006695	cholesterol biosynthetic process	7.9E-6	76.0E-9	23.6	11	31.25	5.00	Specific for 1 day	89.82	17.96
GO:1902653	secondary alcohol biosynthetic process	12.0E-6	76.0E-9	23.6	11	27.78	5.00	Specific for 1 day	89.82	17.96
GO:1901615	organic hydroxy compound metabolic process	30.0E-6	76.0E-9	23.6	11	7.69	10.00	Specific for 1 day	86.42	19.21
GO:0044283	small molecule biosynthetic process	54.0E-6	76.0E-9	23.6	11	5.85	12.00	Specific for 1 day	68.82	45.88
GO:0046165	alcohol biosynthetic process	92.0E-6	76.0E-9	23.6	11	17.86	5.00	Specific for 1 day	89.82	17.96
GO:0006066	alcohol metabolic process	330.0E-6	76.0E-9	23.6	11	8.33	7.00	Specific for 1 day	80.21	26.74
GO:0016053	organic acid biosynthetic process	990.0E-6	76.0E-9	23.6	11	5.97	8.00	Specific for 5 days	54.20	65.04
GO:0046394	carboxylic acid biosynthetic process	990.0E-6	76.0E-9	23.6	11	5.97	8.00	Specific for 5 days	54.20	65.04
GO:1901607	alpha-amino acid biosynthetic process	1.0E-3	76.0E-9	23.6	11	10.42	5.00	Specific for 5 days	49.63	66.17
GO:0008652	cellular amino acid biosynthetic process	1.1E-3	76.0E-9	23.6	11	9.80	5.00	Specific for 5 days	49.63	66.17
GO:0072330	monocarboxylic acid biosynthetic process	14.0E-3	76.0E-9	23.6	11	5.97	4.00	Both times	58.03	58.03
GO:0031331	positive regulation of cellular catabolic process	16.0E-3	76.0E-9	23.6	11	5.71	4.00	Specific for 5 days	43.07	64.60
GO:0009896	positive regulation of catabolic process	17.0E-3	76.0E-9	23.6	11	5.48	4.00	Specific for 5 days	43.07	64.60
GO:0097164	ammonium ion metabolic process	27.0E-3	76.0E-9	23.6	11	6.25	3.00	Specific for 1 day	79.25	26.42
GO:0001878	**Response to yeast**	3.1E-6	4.8E-6	17.7	5	25.00	6.00	Both times	50.00	50.00
GO:0009620	response to fungus	8.6E-6	4.8E-6	17.7	5	20.69	6.00	Both times	50.00	50.00
GO:0031099	**Regeneration**	2.1E-3	150.0E-6	12.7	8	4.94	8.00	Specific for 1 day	75.88	43.36
GO:0042060	wound healing	2.7E-3	150.0E-6	12.7	8	4.68	8.00	Specific for 1 day	73.22	52.30
GO:0042246	tissue regeneration	42.0E-3	150.0E-6	12.7	8	4.08	4.00	Specific for 1 day	77.37	38.69
GO:0007599	hemostasis	44.0E-3	150.0E-6	12.7	8	5.08	3.00	Specific for 5 days	45.51	68.26
GO:0007596	blood coagulation	44.0E-3	150.0E-6	12.7	8	5.08	3.00	Specific for 5 days	45.51	68.26
GO:0050817	coagulation	49.0E-3	150.0E-6	12.7	8	4.84	3.00	Specific for 5 days	45.51	68.26
GO:0006979	**Response to oxidative stress**	2.9E-3	3.9E-3	8.0	0	7.25	5.00	Specific for 1 day	60.00	40.00
GO:0031589	**Cell-substrate adhesion**	8.6E-3	10.0E-3	6.6	3	7.02	4.00	Specific for 5 days	43.07	64.60
GO:0009266	**Response to temperature stimulus**	15.0E-3	19.0E-3	5.7	2	7.89	3.00	Specific for 1 day	100.00	0.00
GO:0000188	**Inactivation of MAPK activity**	230.0E-6	29.0E-3	5.1	10	22.22	4.00	Specific for 5 days	38.69	77.37
GO:0043407	negative regulation of MAP kinase activity	400.0E-6	29.0E-3	5.1	10	19.05	4.00	Specific for 5 days	38.69	77.37
GO:0043409	negative regulation of MAPK cascade	1.0E-3	29.0E-3	5.1	10	14.29	4.00	Specific for 5 days	38.69	77.37
GO:0043405	regulation of MAP kinase activity	1.4E-3	29.0E-3	5.1	10	7.32	6.00	Specific for 5 days	28.72	86.17
GO:0071901	negative regulation of protein serine/threonine kinase activity	1.4E-3	29.0E-3	5.1	10	12.50	4.00	Specific for 5 days	38.69	77.37
GO:0033673	negative regulation of kinase activity	1.9E-3	29.0E-3	5.1	10	6.67	6.00	Specific for 5 days	54.36	67.96
GO:0006469	negative regulation of protein kinase activity	1.9E-3	29.0E-3	5.1	10	6.74	6.00	Specific for 5 days	54.36	67.96
GO:0051348	negative regulation of transferase activity	2.5E-3	29.0E-3	5.1	10	6.25	6.00	Specific for 5 days	54.36	67.96
GO:0001933	negative regulation of protein phosphorylation	2.7E-3	29.0E-3	5.1	10	6.12	6.00	Specific for 5 days	54.36	67.96
GO:0042326	negative regulation of phosphorylation	2.8E-3	29.0E-3	5.1	10	6.00	6.00	Specific for 5 days	54.36	67.96
GO:1902532	negative regulation of intracellular signal transduction	7.0E-3	29.0E-3	5.1	10	4.96	6.00	Specific for 5 days	54.36	67.96
GO:0071900	regulation of protein serine/threonine kinase activity	7.1E-3	29.0E-3	5.1	10	4.92	6.00	Specific for 5 days	28.72	86.17
GO:0001706	endoderm formation	15.0E-3	29.0E-3	5.1	10	8.11	3.00	Specific for 5 days	26.42	79.25
GO:0001704	formation of primary germ layer	17.0E-3	29.0E-3	5.1	10	5.48	4.00	Specific for 5 days	21.53	86.14
GO:0001945	**Lymph vessel development**	37.0E-3	39.0E-3	4.7	4	5.56	3.00	Specific for 1 day	68.26	45.51
GO:0009142	**Nucleoside triphosphate biosynthetic process**	44.0E-3	44.0E-3	4.5	1	5.08	3.00	Specific for 5 days	0.00	100.00
GO:0042541	**Hemoglobin biosynthetic process**	1.2E-3	62.0E-3	4.0	6	23.08	3.00	Specific for 5 days	26.42	79.25
GO:0020027	hemoglobin metabolic process	1.4E-3	62.0E-3	4.0	6	21.43	3.00	Specific for 5 days	26.42	79.25
GO:0055076	transition metal ion homeostasis	49.0E-3	62.0E-3	4.0	6	4.84	3.00	Specific for 5 days	26.42	79.25

**Table 10:** Enrichment of KEGG pathways by the genes differentially expressed after 1 day or 5 days (analyses “1d” vs “5d”). Significantly enriched pathways (FDR < 0.05), identified by their KEGG identifier, are grouped according to 7 functionally related groups obtained by ClueGO analysis.

KEGG Identifier	KEGG ID FDR	Group FDR	Enrichment Score	Groups	% Associated Genes	Nr. Genes	Time	%Genes Specific for 1d	%Genes Specific for 5d
p53 signaling pathway	11.0E-3	11.0E-3	6.5	5	6.76	5.00	Both times	53.89	53.89
Steroid biosynthesis	210.0E-9	210.0E-9	22.2	0	33.33	7.00	Specific for 1d	100.00	0.00
ECM-receptor interaction	1.3E-3	1.3E-3	9.6	6	8.64	7.00	Specific for 1d	66.84	40.11
Alanine, aspartate and glutamate metabolism	7.6E-3	7.6E-3	7.0	1	10.00	4.00	Specific for 1d	64.60	43.07
DNA replication	8.4E-3	8.4E-3	6.9	3	10.53	4.00	Specific for 5 d	25.00	75.00
Arachidonic acid metabolism	12.0E-3	12.0E-3	6.4	2	7.84	4.00	Specific for 5 d	25.00	75.00
Proteasome	14.0E-3	14.0E-3	6.2	4	7.14	4.00	Specific for 5 d	21.53	86.14

## 2 Experimental design, materials, and methods

### 2.1 Experimental set-up and sampling

The experimental set-up generating the analysed RNAs has been previously described by Pinto et al. [1,2]. Immature sea bass (n = 10/experimental group) received intraperitoneal injections of 5 mg/kg E2 or 5 mg/kg Gen in coconut oil or injection of coconut oil alone (control groups). Individual scales were plucked with forceps from the same region of the skin (below the dorsal fin) in each fish, frozen in liquid nitrogen and stored at −80 °C until total RNA extraction.

### 2.2 Total RNA extraction

An automated Maxwell 16 Instrument and the SEV (standard elution volume) total RNA purification kit (Promega, Madison, Wisconsin, USA) were used for the extraction of total RNA from n = 15 scales/individual sea bass. Scales in lysis buffer were mechanically disrupted with an Ultra Turrax homogenizer (IKA, Staufen, Germany) and a dispersing element specialized for fibrous tissues. The extracted total RNA was concentrated by precipitation with 2 vol (V) of 100% ethanol and 1/10 V of 3 M sodium acetate pH 5.2 and resuspended in 20–30 μl of Milli-Q filtered water.

RNA quantity and quality were measured in a NanoDrop 1000 spectrophotometer (Thermo Fisher Scientific, Waltham, Massachusetts, USA). Between 3 and 10 μg of RNA were digested with DNase, using a rigorous treatment regime by carrying out two sequential digestions of 30 min with 2U of TURBO DNAse, as recommended in the instructions of the DNA-free kit (Ambion, Thermo Fisher Scientific, Waltham, Massachusetts, USA).

### 2.3 RNA-seq library preparation

The prepared DNase treated RNAs were precipitated with 5 vol of 100% ethanol and shipped in refrigerated conditions to the Shanghai Ocean University Sequencing Service, Shanghai, China. Their quality was assessed using a Bioanalyser 2100 (Agilent Technologies, Santa Clara, California, USA), to confirm that all RNAs had an RNA integrity number (RIN) greater than 8. Library preparation was conducted using a TruSeq mRNA library prep kit (Illumina, San Diego, California, USA), following the suppliers' instructions. For each library, 0.5 μg of each of the 30 individual RNAs were used (n = 4–6 individual libraries per treatment, see Table 1). Sequencing of paired-end (100 bp) reads was carried out using an Illumina Hi-Seq 1500, at the Shanghai Ocean University Sequencing Service.

### 2.4 Sequencing and data analysis

Quality control and trimming of the produced reads was carried out with FastQC and Cutadapt [3,4], using a Phred quality score cut-off of 20.

Good quality (filtered) reads (Table 1) were then mapped and assembled using the sea bass reference genome assembly from June 2012 (dicLab v1.0c) and the annotation from July 2013 [5,6], by running TopHat and Cufflinks packages with the data and using the default parameters [7,8]. Relative expression levels were obtained in fpkm (fragments per kilobase of transcript per million fragments mapped) for each sea bass gene and differential expression between experimental conditions was evaluated using Cuffdiff.

Differential expression was evaluated using pairwise comparisons between a) the scales of E2-or Gen-treated fish compared to control fish at the same sampling time (E1d *vs* C1d, Gen1d *vs* C1d, E5d *vs* C5d or Gen5d *vs* C5d) or b) between the control groups over time (C1d vs C5d). Two different stringency conditions were used to identify differentially expressed genes: those passing the condition FDR <0.05 and those satisfying both conditions FDR <0.05 and a ≥ 2-fold change in expression between the two compared groups.

The common or specific genes identified between different lists of E2 or Gen DE genes were represented by area proportional Venn diagrams generated with BioVenn [9,10], using their Cufflinks XLOC identifiers (see Supplementary Table 1). Similarities between global transcriptome changes identified for the six experimental conditions (C1d, E1d, Gen1d, C5d, E5d and Gen5d) were evaluated by hierarchical clustering with Cluster 3.0 [11,12], using median centered expression data after Log2 transformation of normalized gene expression (fpkm) levels. The uncentered correlation option and complete linkage options were used to cluster group arrays (experimental conditions).

### 2.5 Gene and functional annotations

The 749 genes for which differential expression was detected with FDR <0.05 were subjected to a multistep automatic gene annotation strategy. Stand-alone Blastx analyses of the corresponding Cufflinks transcripts (individual sequences extracted from the sea bass annotated genes coding sequences, between the mapping positions) were run against the Swiss-Prot curated protein database [13,14] and the unreviewed GenBank protein database [15,16], with expect values (E) set at a maximum of 10^−10^. These Blastx results were then combined and compared with the Cufflinks mappings to the annotated sea bass genome [5,6]. For genome mapping, the annotation of the closest gene was used as long as a maximum distance of 1000 bp existed between this gene and the mapping position of the Cufflinks transcript.

A hierarchical preference order was defined to assign annotation matches to the differentially expressed genes, which was Swiss-Prot hits > sea bass genome hits > GenBank hits, as represented in Fig. 1. Genes were annotated using Swiss-Prot hits for all genes that gave significant hits with this curated database; when no Swiss-Prot hits were found genes were annotated using genome mapping and genes with no significant annotation using the two previous databases were annotated using the non-curated Genbank protein database.

In addition, 54% of the 332 DE genes with ≥ 2-fold change were manually curated to verify the accuracy of the annotations. For this process, individual Cufflinks transcript sequences extracted from the mapping positions were re-annotated using individual BLAT versus the sea bass genome [5,6], compared with the sea bass annotations of these and close by genes. Their predicted coding sequences were blasted against the Swiss-Prot and GenBank protein databases, followed by a careful verification by multisequence alignments carried out with MultAlin [17,18].

Gene ontology (GO) and pathway (KEGG) enrichment analyses were carried out using Cytoscape v3.5.1 [19] and ClueGO plug-in v2.3.2 [20], with Cluepedia v1.3.2. The zebrafish (*Danio rerio*) orthologues for the 371 genes found to be DE with E_2_ or Gen treatments at FDR<0.05 were identified using stand-alone BlastX (with E value < 10^−10^) against the Ensembl zebrafish protein predictions (GRC Zebrafish Build 10, INSDC Assembly GCA_000002035.3 from Sep 2014, downloaded in Feb 2017 at https://www.ensembl.org/ [21]). *D. rerio* Ensembl protein IDs corresponding to each list of DE genes (“All” = all genes regulated by E_2_ and/or Gen; “E_2_” or “Gen” = genes regulated by each treatment irrespective of the sampling time and “1d” or “5d” = genes regulated after 1 or 5 days by either E_2_ or Gen) were then submitted to the Cytoscape/ClueGO plug-in. This was run using the following settings: enrichment analysis (right-sided hypergeometric test) using GO Biological Process (GO BP, levels 3–8) terms for *D. rerio* updated on 13/05/2017; Benjamini–Hochberg false discovery rate (FDR) correction with only terms with FDR ≤ 0.05 and a minimum of three genes/4% being considered significant; Initial group size (for grouping into functionally related networks of enriched terms) set as 1 and group merging at 50%, with a Kappa-statistics score threshold set at 0.4. The enrichment score for each functionally related network group was calculated as -Log2 (group FDR). The leading terms for each group were selected based on their highest enrichment score (lowest term FDR) and were used for group naming in tables and condensed bar plot representations and for evaluation of treatment specific enrichment. Parallel enrichment analyses were carried out for the Kyoto Encyclopaedia of Genes and Genomes (KEGG) pathways for each DE list, using the same parameters and strategy as described for GO BP.

## Footnotes

Appendix A Supplementary data to this article can be found online at https://doi.org/10.1016/j.jsbmb.2019.105448.
